# Matched-Case Comparisons in a Single Institution to Determine Critical Points for Inexperienced Surgeons’ Successful Performances of Laparoscopic Radical Hysterectomy versus Abdominal Radical Hysterectomy in Stage IA2-IIA Cervical Cancer

**DOI:** 10.1371/journal.pone.0131170

**Published:** 2015-06-25

**Authors:** Dong Hoon Suh, Hye-Yon Cho, Kidong Kim, Jae Hong No, Yong-Beom Kim

**Affiliations:** 1 Department of Obstetrics and Gynecology, Seoul National University Bundang Hospital, Seongnam, Korea; 2 Department of Obstetrics and Gynecology, Hallym University Dongtan Sacred Heart Hospital, Hwasung, Korea; University of Texas MD Anderson Cancer Center, UNITED STATES

## Abstract

This is a retrospective study which aims to identify major determinants of successful laparoscopic radical hysterectomy (LRH) versus abdominal radical hysterectomy (ARH) performed by inexperienced surgeons for stage IA2-IIA cervical cancer. A total of 161 consecutive patients with stage IA2–IIA cervical cancer who underwent RH were grouped into 2 groups according to the surgeons’ experience with LRH: experienced surgeon versus inexperienced surgeon. After matching for age and risk factors, surgical and survival outcomes were compared. Experienced surgeon selected patients with earlier-stage and fewer risk factors for LRH than ARH, but inexperience surgeons did not. After matching, the vaginal tumor-free margin of LRH was shorter than that of ARH in experienced surgeon group (1.3 versus 1.7 cm, p=0.007); however, the vaginal tumor-free margin was longer than that of ARH in the inexperienced surgeon group (1.8 versus 1.3 cm, p=0.035). The postoperative hospital stay of LRH was shorter than that of ARH in experienced surgeon group (5.5 versus 7.7 days, p<0.001), but not different from that of ARH in the inexperienced surgeon group. Vaginal tumor-free margin >1.8 cm (OR 7.33, 95% CI 1.22–40.42), stage >IB1 (OR 8.83, 95% CI 1.51–51.73), and estimated blood loss >575 mL (OR 33.95, 95% CI 4.87–236.79) were independent risk factors for longer postoperative hospital stay in the inexperienced surgeon group. There was no difference of 5-year-profression-free survival of LRH patients between experienced surgeon and inexperienced surgeon groups after matching (55.1 versus 33.3%, p=0.391). Selection of earlier-stage disease and moderate vaginal tumor-free margin might be important for an inexperienced surgeon to successfully perform LRH with minimal complications in stage IA2–IIA cervical cancer.

## Introduction

Cervical cancer is the second most common cancer and the third most common cause of cancer deaths in women worldwide [1].

Radical hysterectomy (RH) with pelvic and/or para-aortic lymph node (LN) dissection is a standard primary treatment for early-stage cervical cancer [2]. Although a laparoscopic approach is not yet incorporated into the treatment guidelines, laparoscopic radical hysterectomy (LRH) has become increasingly popular among gynecologic oncologists based on growing evidence supporting its safety and feasibility with equivalent oncologic outcomes of LRH compared with abdominal radical hysterectomy (ARH) for early-stage cervical cancer [3,4].

It is assumed that studies comparing LRH versus ARH might be based on the premise that LRH had been performed by an experienced surgeon, especially for cases with bulky tumor [1]. LRH is a technically challenging procedure, and inexperienced surgeon must overcome a steep learning curve to achieve surgical competency for LRH [5–7]. It is known that at least 40–50 cases are required to reach a turning point in the learning curve of LRH where the operation time and the performance are thought acceptable [3,5].

A phase III randomized controlled trial comparing LRH with ARH in patients with early-stage cervical cancer is currently underway without any prerequisites of surgeon’s experience and competency [8]. It appears inevitable that surgeons at the initial phase of the learning curve will perform LRH for a certain portion of patients who are randomized to the LRH arm in this trial. The impact of surgeons’ experience and competency on the comparison between LRH and ARH should be considered, because several reports have demonstrated learning curve effects on LRH outcome in early-stage cervical cancer [3,5,6]. To our knowledge, however, comparing LRH to ARH according to surgeons’ experience in patients with early-stage cervical cancer has never been reported. The aims of this study were to compare survival and surgical outcomes in LRH versus ARH according to surgeon’s experience of LRH and to identify any determinants of successful LRH for an inexperienced surgeon operating on patients with stage IA2–IIA cervical cancer.

## Materials and Methods

The Institutional Review Board of SNUBH approved this study (B-1312/230-107). Because this was a retrospective study, written informed consent could not be given by the patients for their clinical records to be used in this study. Instead, information of the patient records was anonymized and de-identified prior to analysis.

### Study population

**Fig 1:**
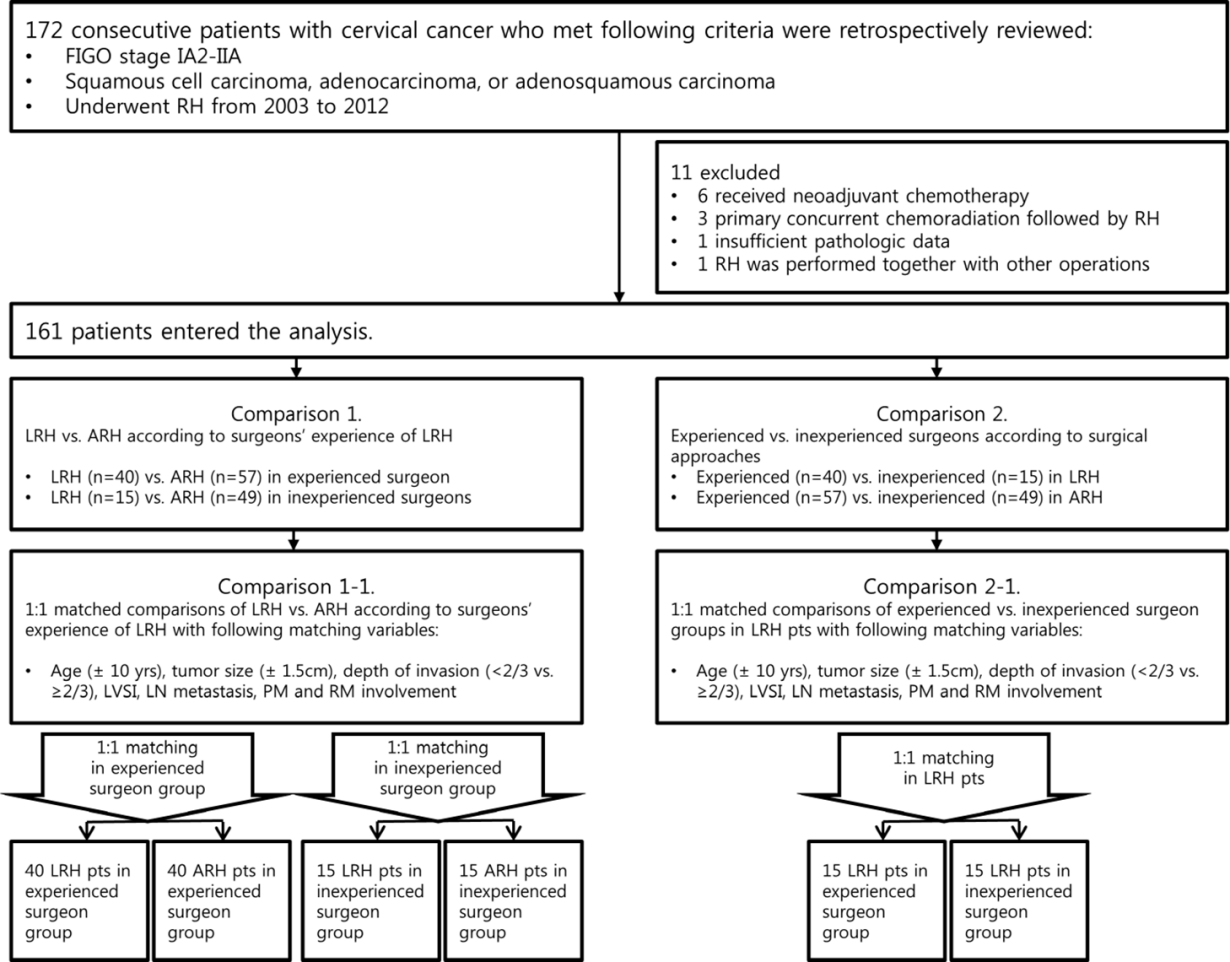
Study design. ARH, abdominal radical hysterectomy; FIGO, the International Federation of Gynecology and Obstetrics; LVSI, lymphovascular space invasion; LN, lymph node; PM, parametrium; pts; patients; RH, radical hysterectomy; RM, resection margin.

We identified 176 patients with FIGO stage IA2 to IIA cervical cancer who underwent RH from 2003 to 2011 at Seoul National University Bundang Hospital (SNUBH) (Fig 1). Fifteen patients were excluded from this study for the following reasons: 4 patients with histologic types other than squamous cell carcinoma, adenocarcinoma, or adenosquamous carcinoma; 9 patients who received neoadjuvant chemotherapy or primary concurrent chemoradiation therapy (CCRT) before RH; 1 who had insufficient clinicopathologic data; 1 who underwent lung lobectomy due to lung cancer at the same time as RH. There was no case of laparoconversion. A final total of 161 consecutive patients entered the analyses, including a matched comparison, after a full retrospective review of the medical records (Table 1).

**Table 1:** Clinicopathologic characteristics and surgical outcomes of LRH versus ARH according to surgeons’ experience of LRH (n = 161)

Characteristics	Experienced		P	Inexperienced		P
	LRH (n = 40)	ARH (n = 57)		LRH (n = 15)	ARH (n = 49)	
Age (years)	48.2±11.5	48.0±11.8	0.934	50.1±11.1	49.4±10.1	0.814
BMI (kg/m^2^)	22.8±2.7	23.2±3.6	0.559	23.4±3.8	23.9±3.8	0.616
Menopause	12 (30.0)	21 (36.8)	0.484	8 (53.3)	20 (40.8)	0.393
FIGO stage			<0.001			0.392
IA2-IB1	39 (97.5)	33 (57.9)		11 (73.3)	30 (61.2)	
IB2-IIA	1 (2.5)	24 (42.1)		4 (26.7)	19 (38.8)	
Tumor size (cm)	2.3±1.2	3.9±1.8	<0.001	2.4±1.9	4.1±2.1	0.005
Large tumor size			0.001			0.001
≤2 cm	19 (47.5)	9 (15.8)		9 (60.0)	7 (14.3)	
>2 cm	21 (52.5)	48 (84.2)		6 (40.0)	42 (85.7)	
Stromal invasion (mm)	7.2±5.0	13.1±6.5	<0.001	8.9±6.4	12.9±6.8	0.051
Deep stromal invasion			<0.001			0.875
≤2/3	31 (81.6)	25 (44.6)		8 (53.3)	25 (51.0)	
>2/3	7 (18.4)	31 (55.4)		7 (46.7)	24 (49.0)	
LVSI			0.381			0.039
Absent	26 (65.0)	32 (56.1)		11 (73.3)	21 (42.9)	
Present	14 (35.0)	25 (43.9)		4 (26.7)	28 (57.1)	
Parametrial involvement			0.069			0.424
Absent	37 (92.5)	45 (78.9)		13 (86.7)	39 (79.6)	
Present	3 (7.5)	12 (21.1)		2 (13.3)	10 (20.4)	
Lymph node metastasis			<0.001			0.128
Absent	37 (92.5)	34 (59.6)		13 (86.7)	33 (67.3)	
Present	3 (7.5)	23 (40.4)		2 (13.3)	16 (32.7)	
Resection margin involvement			0.039			0.667
Absent	40 (100.0)	50 (87.7)		14 (93.3)	46 (93.9)	
Present	0 (0)	7 (12.3)		1 (6.7)	3 (6.1)	
Adjuvant treatment			<0.001			0.047
No	29 (72.5)	17 (29.8)		12 (80.0)	25 (51.0)	
Yes	11 (27.5)	40 (70.2)		3 (20.0)	24 (49.0)	
Vaginal tumor-free margin (cm)	1.3±0.7	1.7±0.8	0.011	1.9±0.7	1.6±1.0	0.697
Nodal yield	21.9±8.3	29.6±11.1	<0.001	22.4±12.1	39.8±59.4	0.266
Operating time (min)	186.5±37.1	196.0±43.0	0.261	250.1±50.6	204.0±57.4	0.007
Estimated blood loss (ml)	293.0±130.1	603.5±311.7	<0.001	616.7±371.1	732.9±353.0	0.275
Postoperative hospital stay (days)	5.5±2.6	7.7±5.2	<0.001	11.2±11.2	10.1±4.8	0.708
Intraoperative ureter injury	0	0	NA	6 (40.0)	2 (4.1)	0.001
Postoperative complication						
Bladder dysfunction	5 (12.5)	7 (12.3)	1.000	5 (33.3)	10 (20.4)	0.241
Lymphedema	11 (27.5)	18 (31.6)	0.666	5 (33.3)	7 (14.3)	0.104
Ureter stricture	1 (2.5)	2 (3.5)	1.000	4 (26.7)	3 (6.1)	0.047
Febrile morbidity*	0	0	NA	1 (6.7)	2 (4.1)	0.558
Wound dehiscence†	0	0	NA	2 (13.3)	1 (2.0)	0.134
Ileus‡	0	0	NA	1 (6.7)	1 (2.0)	0.417
Urinary tract infection	0	0	NA	1 (6.7)	1 (2.0)	0.417
Deep vein thrombosis	1 (2.5)	0	0.412	0	0	NA
Fecal incontinence	0	0	NA	1 (6.7)	0	0.234
Ureterovaginal fistula	0	0	NA	1 (6.7)	0	0.234
Vasovagal syncope	0	0	NA	0	1 (2.0)	0.766

Values are presented as number (%) or mean ± standard deviation.

*as body temperature > 38°C at least for 48 hours after the first 24 hours of surgery;

^†^when it was the only cause of admission or resuture was performed under anesthesia;

^‡^when the patient was treated with Levin-tube insertion or bowel resection and anastomosis. ARH, abdominal radical hysterectomy; BMI, body mass index; FIGO, the International Federation of Gynecology and Obstetrics; LVSI, lymphovascular space invasion; NA, not available.

### Matched comparisons

The RHs were performed by 4 gynecologic oncologic surgeons in SNUBH (S1 Table). They were grouped into 2 groups according to surgeons’ LRH experience, based on previous studies’ reports of a learning curve for LRH. The first group, which included those who had performed ≥40 LRH, was composed of 1 experienced surgeon, and the second group, which included those who had performed <40 LRH, was composed of 3 inexperienced surgeons [5,6]. The single experienced surgeon performed 97 cases of RH (40 LRH and 57 ARH). The 3 inexperienced surgeons performed 64 cases of RH (15 LRH and 49 ARH). The 2 main comparisons performed were: LRH versus ARH according to surgeons’ experience of LRH (comparison 1: Tables 1 and 2) and experienced surgeon versus inexperienced surgeon according to surgical approaches (comparison 2: S2 and S3 Tables). For each comparison, a 1:1 matching process was carried out to equalize the risk factors for recurrence between LRH and ARH cases. The matching process also minimized potential confounding factors using the following matching criteria: age at operation (±10 years), intermediate risk factors [tumor size (±1.5 cm), depth of invasion (<2/3 vs. ≥2/3), LVSI], and high risk factors [LN metastasis, parametrial involvement, and vaginal resection margin (RM) involvement]. Every LRH case in each surgeon group was 1:1 matched to an ARH case in the same surgeon group (40 LRH: 40 ARH in experienced surgeon and 15 LRH: 15 ARH in inexperienced surgeon, comparison 1–1). Every LRH patient in the inexperienced surgeon group was 1:1 matched to an LRH patient in the experienced surgeon group (15 LRH in experienced surgeon: 15 LRH in inexperienced surgeon, comparison 2–1) (Fig 1). In the matching process, a greedy algorithm was used to obtain pairs of the patients by randomly selecting a case from the LRH group and experienced surgeon group, and matching it to a control patient in the ARH group and inexperienced surgeon group, respectively. Briefly, the cases of one group were ordered and sequentially matched to the nearest unmatched control, which exactly matched to each case in terms of above mentioned matching criteria. For the case with more than one matched controls, one control was selected by the randomization method provided by the greedy algorithm. There was no case that did not match to any case in the corresponding control group. After completing the matching process, we found that all the baseline characteristics were evenly distributed between the 2 matched groups (Table 2 and S3 Table).

**Table 2:** One-to-one matched comparison of LRH versus ARH according to surgeons’ experience of LRH

Characteristics	Experienced		P	Inexperienced		P
	LRH (n = 40)	ARH (n = 40)		LRH (n = 15)	ARH (n = 15)	
Age (years)	48.2±11.5	45.9±11.2	0.368	48.8±10.2	48.4±6.3	0.895
BMI (kg/m^2^)	22.8±2.7	23.5±2.6	0.282	23.5±3.9	26.1±5.2	0.155
Menopause	12 (30.0)	19 (47.5)	0.108	8 (53.3)	6 (40.0)	0.464
FIGO stage			1.000			0.651
IA2-IB1	39 (97.5)	39 (97.5)		11 (73.3)	13 (86.7)	
IB2-IIA	1 (2.5)	1 (2.5)		4 (26.7)	2 (13.3)	
Tumor size (cm)	2.3±1.2	2.5±1.3	0.553	2.3±2.0	2.6±1.6	0.672
Large tumor size			1.000			0.464
≤2 cm	19 (47.5)	20 (50.0)		9 (60.0)	7 (46.7)	
>2 cm	21 (52.5)	20 (50.0)		6 (40.0)	8 (53.3)	
Stromal invasion (mm)	7.4±4.9	7.9±5.6	0.687	8.3±6.2	7.7±7.8	0.831
Deep stromal invasion			0.656			0.256
≤2/3	31 (81.6)	31 (77.5)		8 (53.3)	11 (73.3)	
>2/3	7 (18.4)	9 (22.5)		7 (46.7)	4 (26.7)	
LVSI			1.000			1.000
Absent	26 (65.0)	26 (65.0)		11 (73.3)	10 (66.7)	
Present	14 (35.0)	14 (35.0)		4 (26.7)	5 (33.3)	
Parametrial involvement			0.615			1.000
Absent	37 (92.5)	39 (97.5)		13 (86.7)	14 (93.3)	
Present	3 (7.5)	1 (2.5)		2 (13.3)	1 (6.7)	
Lymph node metastasis			1.000			1.000
Absent	37 (92.5)	37 (92.5)		13 (86.7)	13 (86.7)	
Present	3 (7.5)	3 (7.5)		2 (13.3)	2 (13.3)	
Resection margin involvement			0.494			1.000
Absent	40 (100.0)	38 (95.0)		14 (93.3)	15 (100)	
Present	0 (0)	2 (5.0)		1 (6.7)	0	
Adjuvant treatment			0.431			1.000
No	29 (72.5)	32 (80.0)		12 (80.0)	11 (73.3)	
Yes	11 (27.5)	8 (20.0)		3 (20.0)	4 (26.7)	
Vaginal tumor-free margin (cm)	1.3±0.7	1.7±0.7	0.007	1.8±0.8	1.3±0.4	0.035
Nodal yield	21.9±8.3	26.5±8.9	0.018	22.5±12.6	30.7±12.3	0.092
Operating time (min)	186.5±37.1	182.4±38.6	0.629	253.7±50.5	207.1±61.7	0.038
Estimated blood loss (ml)	293.0±130.1	535.0±159.4	<0.001	575.0±346.8	686.4±326.5	0.389
Postoperative hospital stay (days)	5.5±2.6	7.7±2.5	<0.001	11.2±11.2	8.1±3.0	0.312
Intraoperative ureter injury	0	0	NA	6 (40.0)	0	0.017
Postoperative complication						
Bladder dysfunction	5 (12.5)	4 (10.0)	1.000	5 (33.3)	1 (6.7)	0.169
Lymphedema	11 (27.5)	5 (12.5)	0.094	5 (33.3)	1 (6.7)	0.169
Ureter stricture	1 (2.5)	6 (15.0)	0.108	4 (26.7)	2 (13.3)	0.651
Febrile morbidity*	0	0	NA	1 (6.7)	0	1.000
Wound dehiscence†	0	0	NA	2 (13.3)	1 (6.7)	1.000
Ileus‡	0	0	NA	1 (6.7)	0	1.000
Urinary tract infection	0	0	NA	1 (6.7)	0	1.000
Deep vein thrombosis	1 (2.5)	0	1.000	0	0	NA
Fecal incontinence	0	0	NA	1 (6.7)	0	1.000
Ureterovaginal fistula	0	0	NA	1 (6.7)	0	1.000
Vasovagal syncope	0	0	NA	0	0	NA

Values are presented as number (%) or mean ± standard deviation.

*as body temperature > 38°C at least for 48 hours after the first 24 hours of surgery;

^†^when it was the only cause of admission or resuture was performed under anesthesia;

^‡^when the patient was treated with Levin-tube insertion or bowel resection and anastomosis. ARH, abdominal radical hysterectomy; BMI, body mass index; FIGO, the International Federation of Gynecology and Obstetrics; LVSI, lymphovascular space invasion; NA, not available.

### Identification of risk factors for perioperative complications

Based on the finding that mean postoperative hospital stay of patients who experienced intra- or perioperative complications was longer than that of those who did not experience complications (12.2 versus 8.4 days, p = 0.013), we considered postoperative hospital stay to be a surrogate marker for fast recovery without serious complications. For each surgeon group, univariate and multivariate analyses were conducted to assess associations between clinicopathologic factors and the risk of long postoperative hospital stay in order to identify independent risk factors for poor recovery. Of note, hospital stay of our study population is longer than that of the corresponding patients in western countries. The difference of hospital stay between Korea and western countries is mainly attributed to very low admission fee and very high coverage by national medical insurance system in Korea. Cancer patients in Korea pay only 5% of the total medical costs including admission fee, and the rest of them is covered by the national medical insurance. Thus, many patients remain hospitalized postoperatively for a considerable period of time and are usually discharged when they can eat and void well with no specific symptoms associated with postoperative complications.

### Treatment strategy and follow-up

In SNUBH, the treatment policy for early-stage cervical cancer is RH followed by tailored adjuvant therapy depending on the pathologic risk factors.

All patients underwent Piver-Rutledge type 2 or type 3 RH and pelvic and/or papa-aortic LN dissection at the surgeon’s discretion. However, there was no consensus among the 4 surgeons regarding the LRH versus ARH selection criteria of LRH. Surgical procedures of LRH were generally identical to those of ARH except for the skin incision and a uterine manipulator, which was routinely used in every LRH case of our study. For LRH, a periumbilical trocar puncture was made, CO_2_ gas was infused, and 3 other trocar punctures were made. Both round ligaments and infundibulopelvic ligaments were coagulated and cut. The left broad ligament was opened, and both paravesical and pararectal spaces were made by blunt dissection. The perivascular fascial sheath surrounding the external iliac vessels was opened and cleaned. The superior iliac, obturator, and hypogastric node-bearing fatty tissues were cleaned off and removed through the 12 mm trocar site using the Endopouch. The left uterine artery was cleaned near its origin, coagulated, and then cut. The left ureter was cleaned of all surrounding tissues dissected from the uterine artery, and separated as much as possible from the bladder using the Harmonic Scalpel (Ethicon Endo-surgery, USA). The same procedure was performed on the right side. The bladder was anteriorly dissected off the cervix and lower uterine segment. The bladder and ureter were pushed further off the vagina until both ureters could be seen entering the bladder. The posterior vaginal wall was dissected off the cervix to the peritoneal reflexion of the cul-de-sac. The vagina was transected at the level of 1.5 to 2 cm lower from the fornices. The vaginal stump was closed with Vicryl suture. The Foley catheter was usually removed 3 to 4 days after surgery according to the extent of parametrial resection and ureter dissection during surgery.

After surgery, patients with 2 or more intermediate risk factors such as LVSI, tumor size >4 cm, and depth of invasion ≥1/2, were recommended for adjuvant radiation therapy (RT). External beam pelvic RT was given to a dose of 50.4 Gy in 28 fractions (1.8 Gy/fraction), once daily, 5 fractions/week over 5.5 weeks. Patients with 1 or more high risk factors such as parametrial involvement, LN metastasis, and vaginal resection margin involvement were recommended for adjuvant CCRT with weekly cisplatin 40mg/m^2^ intravenously over 1–2 hours for 6 cycles. Without complications, patients who underwent LRH and ARH for the treatment of early-stage cervical cancer were routinely discharged at postoperative day 4 and day 7, respectively, according to the patient’s condition. Follow-up schedules included every 3 months for the first 1 year, every 3–6 months for the next 2–3 years, and then every 6–12 months until 5 years after the last day of treatment. At that point, patients were followed-up annually based on patient’s risk of disease recurrence.

### Surgery-related complications

We counted intra- and postoperative complications. Intraoperative complications were defined as any adverse events during the operation including injury of bowel, ureter, bladder, great vessels, or major nerves, which caused considerable perioperative morbidity. Ureter injury was the only category, which met the criteria of intraoperative complication and was included in the final analysis. Postoperative complications were defined as any adverse events documented within 30 days after the operation. Ureteral stricture and lymphedema, however, were counted if they occurred within 90 days after the operation. Febrile morbidity was defined as body temperature >38°C for at least 48 hours after the first 24 hours following surgery. Wound dehiscence was counted when it was the only cause of admission or when resuturing was performed under anesthesia. Postoperative ileus was counted when the patient was treated with Levin-tube insertion or bowel resection and anastomosis. Bladder dysfunction was counted when the patient had residual urine >150 mL on at least 2 occasions 4 hours apart before discharge and required clean intermittent catheterization after discharge.

### Statistical analysis

Surgical and survival outcomes were compared between LRH and ARH in each surgeon group both before and after matching for variables. The same variables were also compared between experienced surgeon and inexperienced surgeon in LRH and ARH cases. Continuous variables were compared using the student t-test, and categorical variables were compared using the chi-square test. Continuous variables were converted into categorical variables using mean value or a cut-off value, which was established through plotting a receiver operation characteristic curve. Progression-free survival (PFS) was calculated and compared using the Kaplan-Meier method with a log rank test. A 2-sided p-value <0.05 indicated statistical significance. SPSS software (version 19.0; SPSS Inc., Chicago, IL) was used for statistical analyses.

## Results

### LRH versus ARH according to surgeons’ experience of LRH (comparison 1)

Mean ages, body mass index, and menopausal status were similar between LRH and ARH in both experienced surgeon and inexperienced surgeon groups (Table 1). In the experienced surgeon group, LRH patients were more likely to have early-stage cancer, have smaller tumor size, have lesser stromal invasion, have fewer LN metastases, and thus have fewer adjuvant treatments than ARH patients. Notably, LRH patients also had fewer vaginal RM involvements, but a shorter vaginal tumor-free margin (1.3 versus 1.7 cm, p = 0.011) than ARH patients in the experienced surgeon group. By contrast, smaller tumor size and lesser LVSI in LRH patients were the only 2 pathologic variables which showed significant difference between the LRH and ARH patients in the inexperienced surgeon group.

Regarding surgical outcomes, mean operating times of LRH and ARH were similar in the experienced surgeon group. In the inexperienced surgeon group, however, mean operating time for LRH was longer than that for ARH (250.1 versus 204.0 minutes, p = 0.007). Less estimated blood loss (EBL) and shorter postoperative hospital stay were observed in the experienced surgeon group’s LRH patients compared to ARH patients, but no difference was seen in the patients of inexperienced surgeon. Mean nodal yield of LRH was lower than that of ARH in experienced surgeon (21.9 versus 29.6, p<0.001), but not in inexperienced surgeon (22.4 versus 39.8, p = 0.266).

There was no difference in surgery-related complications between LRH and ARH in the experienced surgeon group. However, there were more intraoperative ureter injuries (p = 0.001) and postoperative ureter strictures (p = 0.047) in LRH patients compared to ARH patients in the inexperienced surgeon group.

### Matched comparison of LRH versus ARH according to surgeons’ experience of LRH (comparison 1–1)

Table 2 shows the results of 1:1 matched comparisons of LRH versus ARH in each surgeon group. All clinicopathologic variables were evenly distributed between LRH and ARH patients after a matching process. Vaginal tumor-free margin of LRH was shorter than that of ARH in experienced surgeon (1.3 versus 1.7 cm, p = 0.007), whereas, in the inexperienced surgeon group, vaginal tumor-free margin of LRH was longer than that of ARH (1.8 versus 1.3 cm, p = 0.035). Comparable operating times, lesser EBL, and shorter postoperative hospital stay in LRH compared to ARH patients were still observed after matching in the experienced surgeon group. Longer operating time, similar EBL and postoperative hospital stay in LRH compared with ARH were also found after matching in the inexperienced surgeon group. Intraoperative ureter injury was the only complication which occurred more often in LRH than ARH patients after matching in the inexperienced surgeon group (p = 0.017).

### Risk factors for perioperative complications

Mean postoperative hospital stay was 9.2 days (± 5.8 days). Patients who stayed longer than 9.2 days were considered to be experiencing longer postoperative hospital stay and at risk of suffering from perioperative complications. Stage >IB1 (odds ratio [OR] 6.02, 95% confidence interval [CI] 1.26–28.75), tumor-free margin >0.8 cm (OR 7.23, 95% CI 1.50–34.95), and EBL >575 mL (OR 28.36, 95% CI 4.63–173.84) were independent risk factors for longer postoperative hospital stay in the inexperienced surgeon group (Table 3). Stage >IB1 (OR 4.55, 95% CI 1.48–13.98), ARH versus LRH (OR 13.46, 95% CI 3.70–48.94), and EBL >575 mL (OR 3.47, 95% CI 1.09–11.03) were independent risk factors for longer postoperative hospital stay in the experienced surgeon group (S4 Table).

**Table 3:** Association of clinicopathologic factors with the risk of long postoperative hospital stay in inexperienced surgeon group

Characteristics	No.	Univariate analysis	Multivariate analysis	
		OR (95% CI)	OR (95% CI)	P value
FIGO stage				0.025
IA2-IB1	41	Reference	Reference	
IB2-IIA	23	2.03 (0.72–5.72)	6.02 (1.26–28.75)	
Surgical approach				0.140
ARH	49	Reference	Reference	
LRH	15	1.07 (0.34–3.43)	3.70 (0.65–21.09)	
Vaginal tumor-free margin (cm)				0.014
≤ 1.8	37	Reference	Reference	
> 1.8	27	3.54 (1.25–10.03)	7.23 (1.50–34.95)	
LN retrieved (per 1-LN increment)		1.00 (0.99–1.01)	-	
Operating time (per 1-min increment)		1.00 (0.99–1.01)	-	
Estimated blood loss (ml)				<0.001
≤ 575	26	Reference	Reference	
> 575	38	10.58 (3.00–37.24)	28.36 (4.63–173.84)	
Tumor size (cm)				
≤ 2.0	16	Reference	-	
> 2.0	48	1.53 (0.48–4.89)	-	
Deep stromal invasion				
≤ 2/3	33	Reference	-	
> 2/3	31	1.27 (0.48–3.41)	-	
Parametrial involvement				0.435
Absent	52	Reference	Reference	
Present	12	1.26 (0.36–4.43)	2.29 (0.29–18.15)	
Positive LN				0.484
Absent	46	Reference	Reference	
Present	18	2.44 (0.80–7.47)	1.94 (0.30–12.36)	

ARH, abdominal radical hysterectomy; BMI, body mass index; CI, confidence interval; FIGO, the International Federation of Gynecology and Obstetrics; LN, lymph node; OR, odds ratio

### Experienced versus inexperienced surgeons according to surgical approaches (comparison 2)

In the patients who underwent ARH, all the clinicopathologic variables except adjuvant treatment were similar between the experienced surgeon and inexperienced surgeon groups (S2 Table). Vaginal tumor-free margin was also similar between the 2 surgeon groups in ARH (1.7 versus 1.6 cm, p = 0.838). However, EBL was larger in the inexperienced surgeon group than in the experienced surgeon group (732.9 versus 603.5 mL, p = 0.048). The incidence of intraoperative ureter injury in inexperienced surgeon was not greater than that in experienced surgeon (p = 0.211).

In contrast, in the patients who underwent LRH, the experienced surgeon group had more early—stage ratings (p = 0.017) and lesser stromal invasion (p = 0.046) than in the inexperienced surgeon group. Vaginal tumor-free margin of experienced surgeon group patients was significantly shorter than that in the inexperienced surgeon group (1.3 versus 1.9 cm, p = 0.006). Operating time was shorter (186.5 versus 250.1 minutes, p<0.001), and EBL was smaller (293.0 versus 616.7 mL, p = 0.005) in the experienced surgeon than inexperienced surgeon group. Intraoperative ureter injury (p<0.001) and postoperative ureter stricture (p = 0.017) occurred more frequently in the inexperienced surgeon group than in the experienced surgeon.

After matching, all clinicopathologic variables were comparable between the 2 surgeon groups (S3 Table). Shorter operating time (183.5 versus 250.1 minutes, p<0.001) and lesser EBL (306.7 versus 616.7 mL, p = 0.007) in experienced surgeon compared to the inexperienced surgeon group were still observed after matching. Vaginal tumor-free margin of the experienced surgeon group was no longer shorter than that of the inexperienced surgeon (p = 0.059). Intraoperative ureter injury occurred more frequently (p = 0.017) in the inexperienced surgeon group than the experienced surgeon group.

### Survival outcomes

Mean follow-up time was 44 months (range 0–117 months) for the whole study population: 41.3 months for experienced surgeon and 48.2 months for inexperienced surgeon (p = 0.117). Irrespective of surgical approaches of LRH versus ARH, there were no significant differences in 5-year-PFS (85.2% versus 73.5%, p = 0.103) or 5-year-OS (93.5% versus 98.3%, p = 0.704) between the experienced surgeon (n = 97) and inexperienced surgeon group (n = 64). Irrespective of the surgeon groups, there were no significant differences of 5-year-PFS (72.9% versus 82.1%, p = 0.692) or 5-year-OS (100% versus 94.4%, p = 0.218) between the LRH (n = 55) and ARH approaches (n = 106).

**Fig 2:**
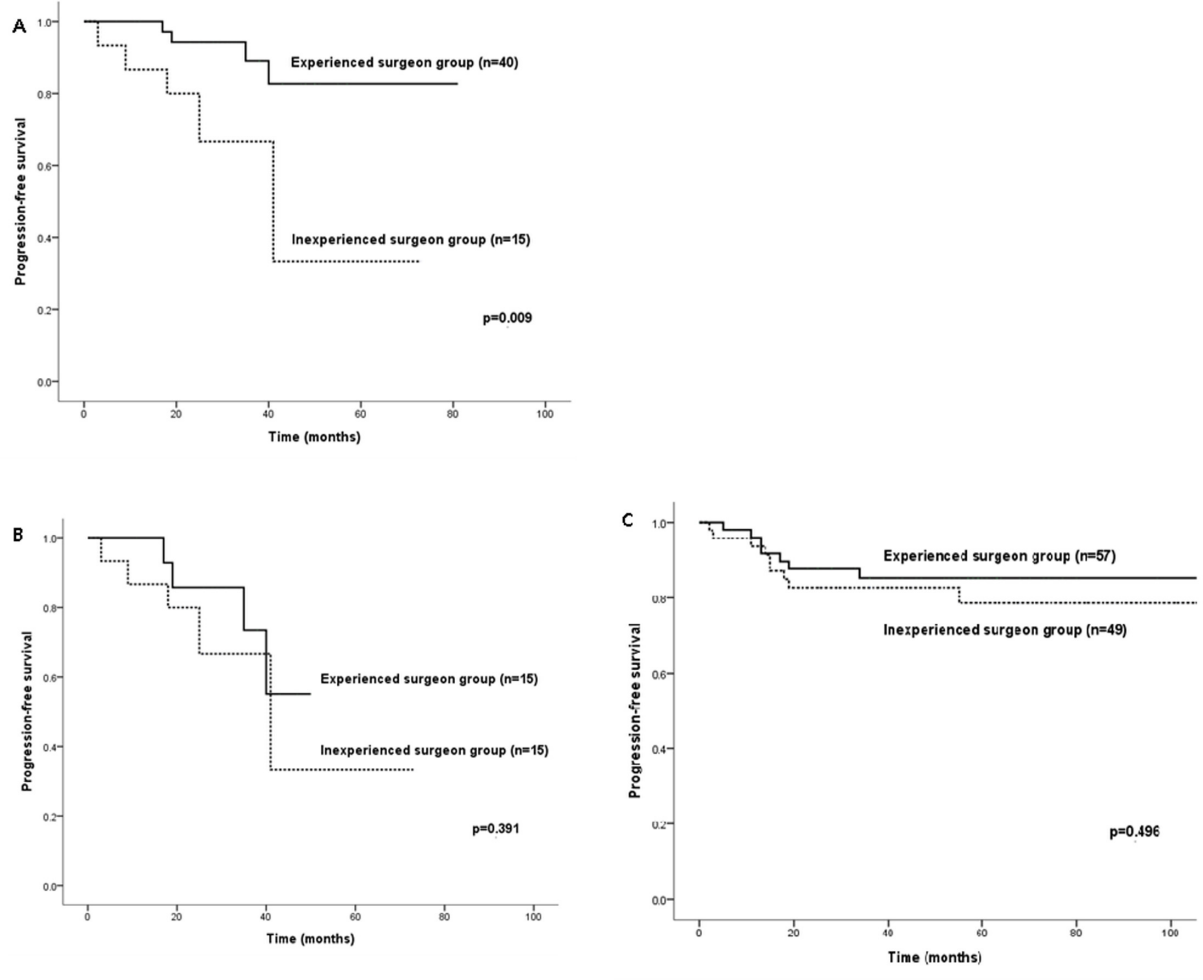
Progression-free survival of patients with early-stage cervical cancer who underwent laparoscopic radical hysterectomy before (A) and after matching (B), and abdominal radical hysterectomy (C) by experienced surgeon group versus inexperienced surgeon group.

There were no significant differences in 5-year-PFS between LRH and ARH even before matching (82.7% versus 85.2%, p = 0.762 for experienced surgeon and 33.3% versus 78.6%, p = 0.155 for inexperienced surgeon) in either the experienced surgeon or inexperienced surgeon groups (comparison 1, S1 Fig). Although 5-year-PFS in the experienced surgeon group was significantly better than that in the inexperienced surgeon group’s LRH patients (82.7% versus 33.3%, p = 0.009) (comparison 2), the statistical significance of the difference disappeared after matching (55.1% versus 33.3%, p = 0.391) (comparison 2–1, Fig 2A and 2B). In ARH patients, however, the 5-year-PFS was not different between experienced surgeon and inexperienced surgeon groups (85.2% versus 78.6%, p = 0.496) (Fig 2C). Survival analysis for OS in LRH patients between experienced surgeon and inexperienced surgeon groups was not performed because there was no mortality in patients who underwent LRH.

## Discussion

We found that the experienced surgeon was more likely to select patients with earlier-stage disease and resect tumors with shorter vaginal tumor-free margin using LRH versus ARH than the inexperienced surgeons. After matching, which equalized stage and risk factors between the 2 surgeon groups, vaginal tumor-free margin of the LRH approach was shorter than that of ARH in the experienced surgeon group, while even longer than that of ARH in the inexperienced surgeon group. Earlier-stage patient selection for LRH and moderate length of vaginal tumor-free margin of the experienced surgeon were significantly associated with lower surgery-related complication than inexperienced surgeon. The LRH versus ARH PFS values were comparable, which just confirmed the findings of the previous relevant studies [9–11]. Although PFS of LRH in the experienced surgeon group was significantly better than that in the inexperienced surgeon group’s LRH patients, statistical significance of the difference disappeared after matching. The clinical implication of our study is not only identifying important determinants of successful LRH for an inexperienced surgeon but also suggesting surgeon’s experience as an important factor to be considered in the design and result analysis of clinical trial of LRH in patients with stage IA2–IIA cervical cancer.

Minimally invasive surgery is an inevitable trend worldwide in surgical treatment of early-stage cervical cancer. Despite the comparable surgical and survival outcomes between experienced surgeon- and inexperienced surgeon-performed ARH, there were many differences of surgical and survival outcomes between experienced surgeon- and inexperienced surgeon-performed LRH in the present study. Experience and surgical competency of surgeons should be considered in studies that compare LRH to ARH as well as studies investigating the learning curve of LRH. Partly due to the difficulty in categorizing the surgeons into experienced surgeon and inexperienced surgeon, to the best of our knowledge, there are no studies which compared LRH versus ARH according to the surgeons’ experience with LRH. We tried to evaluate this intricate issue using rational criteria for surgeon groups (S1 Table) and matched comparisons.

Based on randomized controlled trials, National Comprehensive Cancer Network panel members feel that surgery is the most appropriate option for patients with stage IB1 or IIA1 disease, whereas CCRT is the most appropriate option for those with stage IB2 or IIA2 disease [2]. The authors of the hitherto only study reporting the equivalent therapeutic efficacy and more favorable surgical outcomes of LRH versus ARH in patients with stage IB2 and IIA2 cervical cancer indicated that LRH was believed to be possible in all patients if the surgery was performed by an experienced surgeon [1]. In our study, a stage-equalizing matching process led to similar PFS of LRH patients between experienced surgeon and inexperienced surgeon. Stage >IB1 was a common independent risk factor for a long postoperative hospital stay in both experienced surgeon and inexperienced surgeon groups. Selection of earlier-stage disease for LRH appears to be a prerequisite of successfully performing LRH, especially for an inexperienced surgeon.

Moderate length of tumor-free margin seemed to be another critical point for an inexperienced surgeon to successfully perform LRH in patients with stage IA2–IIA cervical cancer. One possible explanation of longer tumor-free margin of LRH versus ARH in the inexperienced surgeon group after matching could be the inexperienced surgeons’ imperative thought of complete removal of tumor with a sufficient safety margin and only secondary preoccupation with the magnification provided by LRH. An inexperienced surgeon might overreact to the relevant report that the majority of patients who were treated with robotic RH had higher rates of close surgical margin than patients treated with laparotomy (90% versus 58%) [12]. Vaginal tumor-free margin could be affected by the size of the colpotomizer of the uterine manipulator because a uterine manipulator was routinely used in every LRH case of our study. Generally speaking, the larger colpotomizer, the longer vaginal resection margin we have. However, the vaginal resection margin could be short even with a larger colpotomizer, if the surgeon intentionally cut the vagina at the upper level. Moreover, the surgeon himself/herself decides the size of colpotomizer according to the tumor size of uterine cervix. It means that the length of vaginal tumor-free margin totally depends on the surgeon, rather than the size of uterine manipulator. Although few studies have evaluated the prognostic impact of close tumor-free margin in patients who underwent RH for early-stage cervical cancer, debatable close tumor-free margin ranged from 5 mm to 10 mm from the tumor in most previous studies [12–14]. Even a tumor-free margin of ≤5 mm on an RH specimen was not an independent risk factor for disease recurrence [12]. In the present study, mean tumor-free margin of LRH specimens in the inexperienced surgeon group was 1.9 cm ± 0.7 cm, which was obviously long enough for oncologic safety and could be long enough for an inexperienced surgeon to cause unnecessary complications. Nevertheless, long tumor-free margin >1.8 cm was not associated with intraoperative ureter injury in the inexperienced surgeon group. The surgical approach of LRH versus ARH was the only independent risk factor for intraoperative ureter injury in the inexperienced surgeon group (S5 Table).

There were several limitations in our study. The small sample size could have lowered the statistical power. Data from a single institution with a single experienced surgeon might also have prevented the simple generalization of the conclusion. An inherent limitation of a retrospective study was that we could not evaluate every procedure of LRH step-by-step in order to identify critical points of successful LRH in the inexperienced surgeon group. Therefore, we could not identify a specific point of the LRH procedure, which directly affected surgical outcomes, including ureter injury. In order to account for undocumented complications or undiagnosed poor physical condition, which probably delayed early discharge, we considered postoperative hospital stay as a surrogate marker for fast recovery without serious complications.

Despite the limitations, this study is, to our knowledge, the first study to compare survival and surgical outcomes in LRH versus ARH according to surgeon’s experience with LRH and to identify any determinants of successful LRH for inexperienced surgeons in patients with stage IA2–IIA cervical cancer. Matched comparison was performed to control any confounding factors that could affect the results of comparative analyses between LRH and ARH as well as between experienced surgeon and inexperienced surgeon. In conclusion, our study findings suggest that selection of earlier-stage disease and moderate tumor-free margin might be important for an inexperienced surgeon to successfully perform LRH with minimal complication for the treatment of stage IA2–IIA cervical cancer. Further analysis of the coming results of the randomized controlled trial of LRH versus ARH according to the surgeons’ experience of LRH is necessary to confirm the results of this trial [8].

## Supporting Information

S1 Fig Progression-free survival of patients with early-stage cervical cancer who underwent laparoscopic versus abdominal radical hysterectomy by experienced operators (A) and inexperienced operators (B). (TIF)

S1 Table Comparison of the four surgeons in this study according to the surgeons’ experience. (DOCX)

S2 Table Comparison of clinicopathologic characteristics and surgical outcomes between surgeons according to the experience of LRH in LRH and ARH (n = 161). (DOCX)

S3 Table Comparison of clinicopathologic characteristics and surgical outcomes between surgeon groups before and after matching (n = 161). (DOCX)

S4 Table Association of clinicopathologic factors with the risk of long postoperative hospital stay in experienced surgeon group. (DOCX)

S5 Table Association of clinicopathologic factors with the risk of intraoperative ureter injury in inexperienced surgeon group. (DOCX)
