# Supplementary figures and images for: Matched-Case Comparisons in a Single Institution to Determine Critical Points for Inexperienced Surgeons’ Successful Performances of Laparoscopic Radical Hysterectomy versus Abdominal Radical Hysterectomy in Stage IA2-IIA Cervical Cancer
Source: PLoS One. 2015 Jun 25;10(6):e0131170. doi: 10.1371/journal.pone.0131170 (PMC4482442; doi:10.1371/journal.pone.0131170)

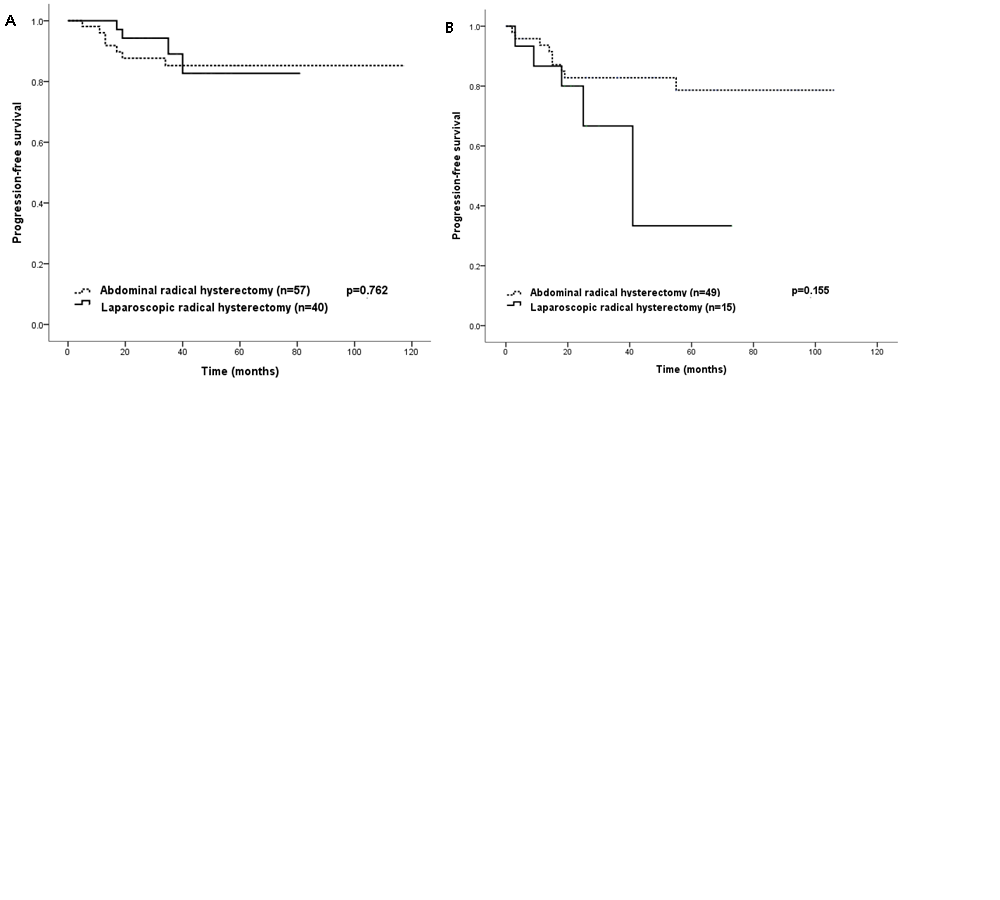

Supplement: S1 Fig — (TIF) [file pone.0131170.s001.tif]
