# Supplementary material for: Matched-Case Comparisons in a Single Institution to Determine Critical Points for Inexperienced Surgeons’ Successful Performances of Laparoscopic Radical Hysterectomy versus Abdominal Radical Hysterectomy in Stage IA2-IIA Cervical Cancer
Source: PLoS One. 2015 Jun 25;10(6):e0131170. doi: 10.1371/journal.pone.0131170 (PMC4482442; doi:10.1371/journal.pone.0131170)
Supplement: S1 Table — (DOCX) [file pone.0131170.s002.docx]

Table S1. Comparison of the four surgeons in this study according to the surgeons’ experience

|  | Experienced | Inexperienced |
| --- | --- | --- |
| Number of operator | 1 | 3 |
| Mean age of operators* | 42 | 38 |
| Years after board* | 9 | 6.7 |
| Previous experience* (mean case number) |  |  |
| LRH | 50 | 5 |
| ARH | >50 | 30 |
| Current study |  |  |
| Number of case | 97 | 64 |
| LRH:ARH ratio | 0.70 (40:57) | 0.31 (15:49) |

*as of the year when they started the first case of LRH in this study. ARH, abdominal radical

hysterectomy; FIGO, the International Federation of Gynecology and Obstetrics; LRH, laparoscopic

radical hysterectomy
