# Supplementary material for: Matched-Case Comparisons in a Single Institution to Determine Critical Points for Inexperienced Surgeons’ Successful Performances of Laparoscopic Radical Hysterectomy versus Abdominal Radical Hysterectomy in Stage IA2-IIA Cervical Cancer
Source: PLoS One. 2015 Jun 25;10(6):e0131170. doi: 10.1371/journal.pone.0131170 (PMC4482442; doi:10.1371/journal.pone.0131170)
Supplement: S2 Table — (DOCX) [file pone.0131170.s003.docx]

Table S2. Comparison of clinicopathologic characteristics and surgical outcomes between surgeons according to the experience of LRH in LRH and ARH (n=161)

|  | LRH | | P | ARH | | P |  |
| --- | --- | --- | --- | --- | --- | --- | --- |
|  | Experienced (n=40) | Inexperienced (n=15) |  | Experienced (n=57) | Inexperienced (n=49) |  |  |
| Age (years) | 48.2±11.5 | 50.1±11.1 | 0.578 | 48.0±11.8 | 49.4±10.2 | 0.516 |  |
| BMI (kg/m^2^) | 22.8±2.7 | 23.4±3.8 | 0.568 | 23.2±3.6 | 23.9±3.8 | 0.334 |  |
| Menopause | 12 (30.0) | 8 (53.3) | 0.109 | 21 (36.8) | 20 (40.8) | 0.675 |  |
| FIGO stage |  |  | 0.017 |  |  | 0.728 |  |
| IA2-IB1 | 39 (97.5) | 11 (73.3) |  | 33 (57.9) | 30 (61.2) |  |  |
| IB2-IIA | 1 (2.5) | 4 (26.7) |  | 24 (42.1) | 19 (38.8) |  |  |
| Tumor size (cm) | 2.3±1.2 | 2.4±1.9 | 0.930 | 3.9±1.8 | 4.1±2.1 | 0.567 |  |
| Large tumor size |  |  | 0.409 |  |  | 0.829 |  |
| ≤2 cm | 19 (47.5) | 9 (60.0) |  | 9 (15.8) | 7 (14.3) |  |  |
| >2 cm | 21 (52.5) | 6 (40.0) |  | 48 (84.2) | 42 (85.7) |  |  |
| Stromal invasion (mm) | 7.2±5.0 | 8.9±6.4 | 0.285 | 13.1±6.5 | 12.9±6.8 | 0.845 |  |
| Deep stromal invasion |  |  | 0.046 |  |  | 0.514 |  |
| ≤2/3 | 31 (81.6) | 8 (53.3) |  | 25 (44.6) | 25 (51.0) |  |  |
| >2/3 | 7 (18.4) | 7 (46.7) |  | 31 (55.4) | 24 (49.0) |  |  |
| LVSI |  |  | 0.749 |  |  | 0.173 |  |
| Absent | 26 (65.0) | 11 (73.3) |  | 32 (56.1) | 21 (42.9) |  |  |
| Present | 14 (35.0) | 4 (26.7) |  | 25 (43.9) | 28 (57.1) |  |  |
| Parametrial involvement |  |  | 0.606 |  |  | 0.935 |  |
| Absent | 37 (92.5) | 13 (86.7) |  | 45 (78.9) | 39 (79.6) |  |  |
| Present | 3 (7.5) | 2 (13.3) |  | 12 (21.1) | 10 (20.4) |  |  |
| Lymph node metastasis |  |  | 0.606 |  |  | 0.413 |  |
| Absent | 37 (92.5) | 13 (86.7) |  | 34 (59.6) | 33 (67.3) |  |  |
| Present | 3 (7.5) | 2 (13.3) |  | 23 (40.4) | 16 (32.7) |  |  |
| Resection margin involvement |  |  | 0.273 |  |  | 0.335 |  |
| Absent | 40 (100.0) | 14 (93.3) |  | 50 (87.7) | 46 (93.9) |  |  |
| Present | 0 | 1 (6.7) |  | 7 (12.3) | 3 (6.1) |  |  |
| Adjuvant treatment |  |  | 0.734 |  |  | 0.026 |  |
| No | 29 (72.5) | 12 (80.0) |  | 17 (29.8) | 25 (51.0) |  |  |
| Yes | 11 (27.5) | 3 (20.0) |  | 40 (70.2) | 24 (49.0) |  |  |
| Vaginal tumor-free margin (cm) | 1.3±0.7 | 1.9±0.7 | 0.006 | 1.7±0.8 | 1.6±1.0 | 0.838 |  |
| Nodal yield | 21.9±8.3 | 22.4±12.1 | 0.855 | 29.6±11.1 | 39.8±59.4 | 0.205 |  |
| Operating time (min) | 186.5±37.1 | 250.1±50.6 | <0.001 | 196.0±43.0 | 204.0±57.4 | 0.426 |  |
| Estimated blood loss (ml) | 293.0±130.1 | 616.7±371.1 | 0.005 | 603.5±311.7 | 732.9±353.0 | 0.048 |  |
| Postoperative hospital stay (days) | 5.2±2.2 | 11.5±11.2 | 0.049 | 10.6±5.2 | 10.2±4.9 | 0.661 |  |
| Intraoperative ureter injury | 0 | 6 (40.0) | <0.001 | 0 | 2 (4.1) | 0.211 |  |
| Postoperative complication |  |  |  |  |  |  |  |
| Bladder dysfunction | 5 (12.5) | 5 (33.3) | 0.115 | 7 (12.3) | 10 (20.4) | 0.256 |  |
| Lymphedema | 11 (27.5) | 5 (33.3) | 0.744 | 18 (31.6) | 7 (14.3) | 0.037 |  |
| Ureter stricture | 1 (2.5) | 4 (26.7) | 0.017 | 2 (3.5) | 3 (6.1) | 0.660 |  |
| Febrile morbidity* | 0 | 1 (6.7) | 0.273 | 0 | 2 (4.1) | 0.221 |  |
| Wound dehiscence† | 2 (13.3) | 0 | 0.071 | 0 | 1 (2.0) | 0.462 |  |
| Ileus‡ | 0 | 1 (6.7) | 0.273 | 0 | 1 (2.0) | 0.462 |  |
| Urinary tract infection | 0 | 1 (6.7) | 0.273 | 0 | 1 (2.0) | 0.462 |  |
| Deep vein thrombosis | 1 (2.5) | 0 | 1.000 | 0 | 0 | NA |  |
| Fecal incontinence | 0 | 1 (6.7) | 0.273 | 0 | 0 | NA |  |
| Ureterovaginal fistula | 0 | 1 (6.7) | 0.273 | 0 | 0 | NA |  |
| Vasovagal syncope | 0 | 0 | NA | 0 | 1 (2.0) | 0.462 |  |

ARH, abdominal radical hysterectomy; BMI, body mass index; FIGO, the International Federation of Gynecology and Obstetrics; LVSI, lymphovascular space invasion.
