# Supplementary material for: Matched-Case Comparisons in a Single Institution to Determine Critical Points for Inexperienced Surgeons’ Successful Performances of Laparoscopic Radical Hysterectomy versus Abdominal Radical Hysterectomy in Stage IA2-IIA Cervical Cancer
Source: PLoS One. 2015 Jun 25;10(6):e0131170. doi: 10.1371/journal.pone.0131170 (PMC4482442; doi:10.1371/journal.pone.0131170)
Supplement: S3 Table — (DOCX) [file pone.0131170.s004.docx]

Table S3. Comparison of clinicopathologic characteristics and surgical outcomes between surgeon groups before and after matching (n=161)

|  | LRH before matching | | P | LRH after matching | | P |  |
| --- | --- | --- | --- | --- | --- | --- | --- |
|  | Experienced (n=40) | Inexperienced (n=15) |  | Experienced (n=15) | Inexperienced (n=15) |  |  |
| Age (years) | 48.2±11.5 | 50.1±11.1 | 0.578 | 49.5±11.3 | 50.1±11.1 | 0.884 |  |
| BMI (kg/m^2^) | 22.8±2.7 | 23.4±3.8 | 0.568 | 22.4±1.8 | 23.4±3.8 | 0.368 |  |
| Menopause | 12 (30.0) | 8 (53.3) | 0.109 | 6 (40.0) | 8 (53.3) | 0.464 |  |
| FIGO stage |  |  | 0.017 |  |  | 0.100 |  |
| IA2-IB1 | 39 (97.5) | 11 (73.3) |  | 15 (100) | 11 (73.3) |  |  |
| IB2-IIA | 1 (2.5) | 4 (26.7) |  | 0 | 4 (26.7) |  |  |
| Tumor size (cm) | 2.3±1.2 | 2.4±1.9 | 0.930 | 2.1±1.1 | 2.4±1.9 | 0.618 |  |
| Large tumor size |  |  | 0.409 |  |  | 1.000 |  |
| ≤2 cm | 19 (47.5) | 9 (60.0) |  | 8 (53.3) | 9 (60.0) |  |  |
| >2 cm | 21 (52.5) | 6 (40.0) |  | 7 (46.7) | 6 (40.0) |  |  |
| Stromal invasion (mm) | 7.2±5.0 | 8.9±6.4 | 0.285 | 8.3±5.6 | 8.9±6.4 | 0.765 |  |
| Deep stromal invasion |  |  | 0.046 |  |  | 0.456 |  |
| ≤2/3 | 31 (81.6) | 8 (53.3) |  | 10 (66.7) | 8 (53.3) |  |  |
| >2/3 | 7 (18.4) | 7 (46.7) |  | 5 (33.3) | 7 (46.7) |  |  |
| LVSI |  |  | 0.749 |  |  | 1.000 |  |
| Absent | 26 (65.0) | 11 (73.3) |  | 10 (66.7) | 11 (73.3) |  |  |
| Present | 14 (35.0) | 4 (26.7) |  | 5 (33.3) | 4 (26.7) |  |  |
| Parametrial involvement |  |  | 0.606 |  |  | 1.000 |  |
| Absent | 37 (92.5) | 13 (86.7) |  | 13 (86.7) | 13 (86.7) |  |  |
| Present | 3 (7.5) | 2 (13.3) |  | 2 (13.3) | 2 (13.3) |  |  |
| Lymph node metastasis |  |  | 0.606 |  |  | 1.000 |  |
| Absent | 37 (92.5) | 13 (86.7) |  | 13 (86.7) | 13 (86.7) |  |  |
| Present | 3 (7.5) | 2 (13.3) |  | 2 (13.3) | 2 (13.3) |  |  |
| Resection margin involvement |  |  | 0.273 |  |  | 1.000 |  |
| Absent | 40 (100.0) | 14 (93.3) |  | 15 (100) | 14 (93.3) |  |  |
| Present | 0 | 1 (6.7) |  | 0 | 1 (6.7) |  |  |
| Adjuvant treatment |  |  | 0.734 |  |  | 0.427 |  |
| No | 29 (72.5) | 12 (80.0) |  | 9 (60.0) | 12 (80.0) |  |  |
| Yes | 11 (27.5) | 3 (20.0) |  | 6 (40.0) | 3 (20.0) |  |  |
| Vaginal tumor-free margin (cm) | 1.3±0.7 | 1.9±0.7 | 0.006 | 1.3±0.7 | 1.8±0.8 | 0.059 |  |
| Nodal yield | 21.9±8.3 | 22.4±12.1 | 0.855 | 22.4±8.6 | 22.4±12.1 | 1.000 |  |
| Operating time (min) | 186.5±37.1 | 250.1±50.6 | <0.001 | 183.5±37.2 | 250.1±50.6 | <0.001 |  |
| Estimated blood loss (ml) | 293.0±130.1 | 616.7±371.1 | 0.005 | 306.7±141.3 | 616.7±371.1 | 0.007 |  |
| Postoperative hospital stay (days) | 5.2±2.2 | 11.5±11.2 | 0.049 | 6.0±3.5 | 11.5±11.2 | 0.081 |  |
| Intraoperative ureter injury | 0 | 6 (40.0) | <0.001 | 0 | 6 (40.0) | 0.017 |  |
| Postoperative complication |  |  |  |  |  |  |  |
| Bladder dysfunction | 5 (12.5) | 5 (33.3) | 0.115 | 2 (13.3) | 5 (33.3) | 0.390 |  |
| Lymphedema | 11 (27.5) | 5 (33.3) | 0.744 | 7 (46.7) | 5 (33.3) | 0.456 |  |
| Ureter stricture | 1 (2.5) | 4 (26.7) | 0.017 | 0 | 4 (26.7) | 0.100 |  |
| Febrile morbidity* | 0 | 1 (6.7) | 0.273 | 0 | 1 (6.7) | 1.000 |  |
| Wound dehiscence† | 2 (13.3) | 0 | 0.071 | 0 | 2 (13.3) | 0.483 |  |
| Ileus‡ | 0 | 1 (6.7) | 0.273 | 0 | 1 (6.7) | 1.000 |  |
| Urinary tract infection | 0 | 1 (6.7) | 0.273 | 0 | 1 (6.7) | 1.000 |  |
| Deep vein thrombosis | 1 (2.5) | 0 | 1.000 | 1 (6.7) | 0 | 1.000 |  |
| Fecal incontinence | 0 | 1 (6.7) | 0.273 | 0 | 1 (6.7) | 1.000 |  |
| Ureterovaginal fistula | 0 | 1 (6.7) | 0.273 | 0 | 1 (6.7) | 1.000 |  |
| Vasovagal syncope | 0 | 0 | NA | 0 | 0 | NA |  |

ARH, abdominal radical hysterectomy; BMI, body mass index; FIGO, the International Federation of Gynecology and Obstetrics; LVSI, lymphovascular space invasion.
