# Supplementary material for: Matched-Case Comparisons in a Single Institution to Determine Critical Points for Inexperienced Surgeons’ Successful Performances of Laparoscopic Radical Hysterectomy versus Abdominal Radical Hysterectomy in Stage IA2-IIA Cervical Cancer
Source: PLoS One. 2015 Jun 25;10(6):e0131170. doi: 10.1371/journal.pone.0131170 (PMC4482442; doi:10.1371/journal.pone.0131170)
Supplement: S4 Table — (DOCX) [file pone.0131170.s005.docx]

Table S4. Association of clinicopathologic factors with the risk of long postoperative hospital stay in experienced surgeon group

| Characteristics | No. | Univariate analysis | Multivariate analysis | |
| --- | --- | --- | --- | --- |
|  |  | OR (95% CI) | OR (95% CI) | P value |
| Age (per 1-year increment) |  | 1.01 (0.98-1.05) |  |  |
| FIGO stage |  |  |  | 0.008 |
| IA2-IB1 | 72 | Reference | Reference |  |
| IB2-IIA | 25 | 8.32 (2.97-23.27) | 4.55 (1.48-13.98) |  |
| Surgical approach |  |  |  | <0.001 |
| LRH | 57 | Reference | Reference |  |
| ARH | 40 | 10.73 (3.37-34.13) | 13.46 (3.70-48.94) |  |
| Vaginal tumor-free margin (cm) |  |  |  | 0.092 |
| ≤ 1.8 | 54 | Reference | Reference |  |
| > 1.8 | 43 | 3.30 (1.39-7.84) | 2.88 (0.84-9.88) |  |
| LN retrieved (per 1-LN increment) |  | 1.07 (1.02-1.12) | 1.01 (0.95-1.07) | 0.847 |
| Operating time (per 1-min increment) |  | 1.01 (1.00-1.02) | 1.01 (0.99-1.03) | 0.243 |
| Estimated blood loss (ml) |  |  |  | 0.035 |
| ≤ 575 | 70 | Reference | Reference |  |
| > 575 | 27 | 8.02 (3.00-21.72) | 3.47 (1.09-11.03) |  |
| Tumor size (cm) |  |  |  | 0.254 |
| ≤ 2.0 | 28 | Reference | Reference |  |
| > 2.0 | 69 | 7.21 (1.99-26.12) | 3.04 (0.45-20.50) |  |
| Deep stromal invasion |  |  |  | 0.514 |
| ≤ 2/3 | 56 | Reference | Reference |  |
| > 2/3 | 38 | 3.00 (1.25-7.22) | 1.67 (0.36-7.77) |  |
| Parametrial involvement |  |  |  | 0.464 |
| Absent | 82 | Reference | Reference |  |
| Present | 15 | 4.56 (1.41-14.72) | 2.15 (0.28-16.56) |  |
| Positive LN |  |  |  | 0.481 |
| Absent | 71 | Reference | Reference |  |
| Present | 26 | 4.38 (1.70-11.31) | 1.76 (0.37-8.44) |  |

ARH, abdominal radical hysterectomy; BMI, body mass index; CI, confidence interval; FIGO, the International Federation of Gynecology and Obstetrics; LN, lymph node; OR, odds ratio
