# Supplementary material for: Matched-Case Comparisons in a Single Institution to Determine Critical Points for Inexperienced Surgeons’ Successful Performances of Laparoscopic Radical Hysterectomy versus Abdominal Radical Hysterectomy in Stage IA2-IIA Cervical Cancer
Source: PLoS One. 2015 Jun 25;10(6):e0131170. doi: 10.1371/journal.pone.0131170 (PMC4482442; doi:10.1371/journal.pone.0131170)
Supplement: S5 Table — (DOCX) [file pone.0131170.s006.docx]

| Characteristics | No. | Univariate analysis | Multivariate analysis | |
| --- | --- | --- | --- | --- |
|  |  | OR (95% CI) | OR (95% CI) | P value |
| Age (per 1-year increment) |  | 0.99 (0.92-1.06) |  |  |
| BMI (kg/m^2^) |  |  |  |  |
| ≤ 30 | 59 | Reference |  |  |
| > 30 | 5 | 0.40 (0.05-3.32) |  |  |
| FIGO stage |  |  |  |  |
| IA2-IB1 | 41 | Reference |  |  |
| IB2-IIA | 23 | 0.22 (0.03-1.92) |  |  |
| Tumor size (per 1 mm increment) |  | 0.55 (0.32-0.95) | 0.50 (0.19-1.34) | 0.169 |
| Parametrial involvement |  |  |  | 0.067 |
| Absent | 52 | Reference | Reference |  |
| Present | 12 | 3.13 (0.63-15.51) | 9.27 (0.86-100.52) |  |
| Number of LN retrieved (per 1 LN increment) |  | 0.89 (0.81-0.98) | 0.92 (0.82-1.03) | 0.156 |
| Vaginal tumor-free margin (cm) |  |  |  |  |
| ≤ 2.2 | 44 | Reference |  |  |
| > 2.2 | 20 | 1.26 (0.24-6.54) |  |  |
| Surgical approach |  |  |  | 0.002 |
| ARH | 49 | Reference | Reference |  |
| LRH | 15 | 15.67 (2.72-90.34) | 15.67 (2.72-90.34) |  |

Table S5. Association of clinicopathologic factors with the risk of intraoperative ureter injury in inexperienced surgeon group

ARH, abdominal radical hysterectomy; BMI, body mass index; CI, confidence interval; FIGO, the International Federation of Gynecology and Obstetrics; LN, lymph node; OR, odds ratio
